# Personalized Medicine in Ocular Fibrosis: Myth or Future Biomarkers

**DOI:** 10.1089/wound.2015.0677

**Published:** 2016-09-01

**Authors:** Cynthia Yu-Wai-Man, Peng Tee Khaw

**Affiliations:** National Institute for Health Research (NIHR) Biomedical Research Centre at Moorfields Eye Hospital NHS Foundation Trust and UCL Institute of Ophthalmology, London, United Kingdom.

**Keywords:** personalized medicine, biomarkers, fibrosis, genotyping, phenotyping

## Abstract

**Significance:** Fibrosis-related events play a part in the pathogenesis or failure of treatment of virtually all the blinding diseases around the world, and also account for over 40% of all deaths. It is well established that the eye and other tissues of some group of patients, for example Afro-Caribbean people, scar worse than others. However, there is a current lack of reliable biomarkers to stratify the risk of scarring and postsurgical fibrosis in the eye.

**Recent Advances:** Recent studies using genomics, proteomics, metabolomics, clinical phenotyping, and high-resolution *in vivo* imaging techniques have revealed potential novel biomarkers to identify and stratify patients at risk of scarring in different fibrotic eye diseases.

**Critical Issues:** Most of the studies, to date, have been done in animals or small cohorts of patients and future research is needed to validate these results in large longitudinal human studies. Detailed clinical phenotyping and effective biobanking of patient tissues will also be critical for future biomarker research in ocular fibrosis.

**Future Directions:** The ability to predict the risk of scarring and to tailor the antifibrotic treatment regimen to each individual patient will be an extremely useful tool clinically to prevent undertreating, or exposing patients to unnecessary treatments with potential side effects. An exciting future prospect will be to use new advances in genotyping, namely next-generation whole genome sequencing like RNA-Seq, to develop a customized gene chip in ocular fibrosis. Successful translation of future biomarkers to benefit patient care will also ultimately require a strong collaboration between academics, pharmaceutical, and biotech companies.

**Figure:**
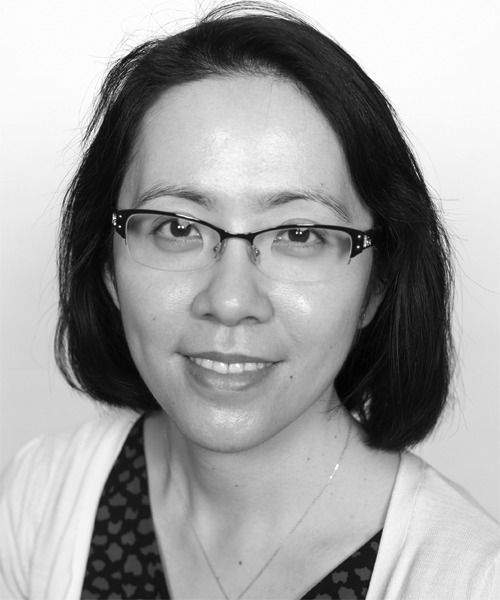
**Cynthia Yu-Wai-Man, MBBS, FRCOphth**

## Scope and Significance

**Figure 1.**
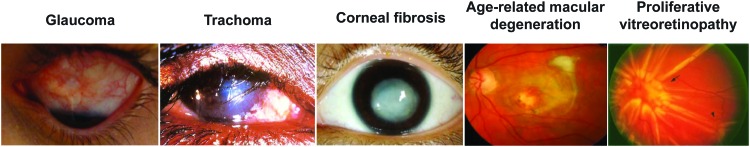
Fibrosis forms part of the pathogenesis or failure of treatment of most blinding diseases worldwide such as glaucoma, trachoma, corneal fibrosis, age-related macular degeneration, and proliferative vitreoretinopathy. To see this illustration in color, the reader is referred to the web version of this article at www.liebertpub.com/wound

Fibrosis-related events play a part in most of the blinding diseases worldwide (Fig. 1) and also account for over 40% of all deaths. It is well established that the eye tissues of some group of patients, for example Afro-Caribbean people, scar worse than others. However, there is a current lack of reliable biomarkers to stratify the risk of scarring and postsurgical fibrosis in the eye. This review focuses on the recent advances in genomics, proteomics, metabolomics, clinical phenotyping, and high-resolution *in vivo* imaging techniques that might help to identify and stratify the groups of patients at risk of scarring in different parts of the eye.

## Translational Relevance

In the next 10 years, the hope is that new advances in genotyping, namely next-generation whole genome sequencing, and detailed clinical phenotyping using modern tissue biomarkers and high-resolution *in vivo* imaging techniques, will help to identify the groups of patients that would scar more aggressively, and thus help to develop a more personalized and stratified approach in antifibrotic ocular therapeutics. Successful translation of future biomarkers in ocular fibrosis will also ultimately require a strong collaboration between academics, pharmaceutical, and biotech companies.

## Clinical Relevance

There is a large unmet clinical need for new predictive and mechanistic biomarkers in ocular fibrosis. Being able to predict patients' risk of scarring and to tailor the antifibrotic treatment regimen to each individual patient will be an extremely useful tool clinically to prevent undertreating, or exposing them to unnecessary treatments with potential side effects. Most of the studies, to date, have been carried out in animals or small cohorts of patients, and future research is thus needed to validate these results in large longitudinal human studies. Detailed clinical phenotyping and effective biobanking of patient tissues will also be critical for future biomarker research in ocular fibrosis.

## Discussion

### Tissue genomics

The NEIBank is a project to gather and organize genomic resources for eye research.^1^ The NEIBank includes expressed sequence tag data and sequence-verified cDNA clones for multiple eye tissues of several species, web-based access to human eye-specific serial analysis of gene expression (SAGE) data through EyeSAGE, and comprehensive annotated databases of known human eye disease genes and candidate disease gene loci.^2–5^

**Figure 2.**
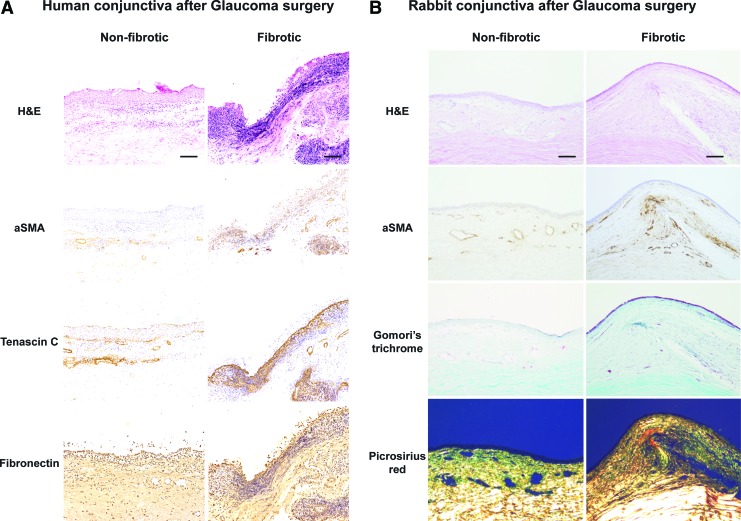
The conjunctiva undergoes marked histopathological changes after glaucoma filtration surgery in **(A)** humans and **(B)** a rabbit model of conjunctival fibrosis. There is increased cellularity and αSMA staining in fibrotic human and rabbit conjunctiva compared to nonfibrotic conjunctiva. Scale bar = 50 μm. αSMA, alpha smooth muscle actin. To see this illustration in color, the reader is referred to the web version of this article at www.liebertpub.com/wound

Glaucoma is the commonest cause of irreversible blindness in the world and conjunctival fibrosis is the major determinant of the surgical success after glaucoma filtration surgery (Fig. 2). Popp *et al.* isolated anterior segment tissues (cornea, conjunctiva, iris) and posterior segment tissues (lens, retina, sclera) of rabbit eyes, and created two independent cDNA libraries through the NEIBank project.^6^ Using microarray analysis, they found the expression of 315 genes to be significantly altered in the rabbit conjunctiva and Tenon's capsule after glaucoma filtration surgery, and these genes included proteins associated with the inflammatory response, defense response, and proteins involved in the synthesis of the extracellular matrix.

Esson *et al.* also performed a microarray analysis of blebs after glaucoma filtration surgery in Sprague Dawley rats and found a significant treatment effect in 923 genes.^7^ Their results confirmed the expression patterns of known mediators of the bleb scarring process, including transforming growth factor-β, connective tissue growth factor, matrix metalloproteinases, and structural proteins like collagens.

In addition, Mahale *et al.* used the human RT^2^ Profiler polymerase chain reaction (PCR) array to study the differential gene expression between seven capsules of failed Ahmed glaucoma valves and two control Tenon's capsules.^8^ They found 39 genes with more than two-fold differential gene expression in three or more of the capsules, including *CTGF, THBS1, SERPINE1, THBS2, COL3A1, MMP3,* and *IL1A* genes. Inflammation is a known risk factor for scarring after glaucoma surgery,^9^ and Mahale *et al.* also found dysregulation of several genes expressing inflammatory cytokines and chemokines (*CCL11*, *IL13*, *IL1A*, *IL1B*, *CXCR4*).^8^ Their results are supported by the work of Chang *et al.* who found increased mast cell numbers in the conjunctival tissues of patients with previous glaucoma surgery that might be associated with an increased risk of conjunctival scarring^10,11^ and previous findings that chronic treatment with eyedrops resulted in an increase in inflammatory cells and an increased chance of surgical failure.^12,13^

Conjunctival scarring is also critical following Chlamydia trachomatis infection that leads to trichiasis (inturned eyelids) and painful blindness in trachoma. Burton *et al.* performed a microarray analysis on 45 patients with trachomatous trichiasis (TT), as well as real-time quantitative PCR for 16 gene expression targets on 386 TT patients and 386 normal controls.^14^ They found that the gene expression profile of TT patients was consistent with squamous metaplasia (keratins, SPRR), proinflammatory cytokine production (*IL1β*, *CXCL5*, *S100A7*), and tissue remodeling (*MMP7*, *MMP9*, *MMP12*, *HAS3*). Clinical inflammation was associated with increased *S100A7*, *IL1B, IL17A, CXCL5, CTGF, CEACAM5, MMP7, CD83* and reduced *SPARCL1* gene expression.^15^ Burton *et al.* also reported an increased expression of the *S100A7* (psoriasin) gene in patients with recurrent TT.^16^

Ocular cicatricial pemphigoid (OCP) is another sight-threatening disease that is associated with severe and chronic conjunctival fibrosis. Razzaque *et al.* compared conjunctival fibroblasts from 10 OCP patients and 5 normal controls. They found an increased expression of *m-CSF*,^17^ *CTGF*,^18^ *HSP47*,^19^ and macrophage migration inhibitory factor (*MIF*)^20^ genes in OCP patients using real-time quantitative PCR. Saw *et al.* also reported an increased expression of interleukin-13^21^ and tumor necrosis factor-alpha^22^ in mucous membrane pemphigoid fibroblasts compared to normal human fibroblasts.

Corneal fibrosis is a leading cause of blindness worldwide and can occur after corneal injury, surgery, or secondary to infection (e.g., herpetic keratitis) or inflammation (e.g., pterygium). Varela *et al.* carried out a microarray analysis on rat corneas after Excimer laser photorefractive keratectomy and identified 73 genes with a three-fold change in expression compared to untreated corneas.^23^ These included genes that play an important role in corneal wound healing, namely growth factors, cell cycle regulators, transcription factors, and metabolic pathway genes.

Using cDNA microarrays, Cao *et al.* also found the expression of 37 genes to be upregulated and that of 27 genes to be downregulated more than five-fold in healing mouse corneas compared to normal uninjured corneas.^24^ The upregulated genes included ICAM-1, macrophage inflammatory proteins, SOCS, IL-10 receptor, and galectin-7. Among the downregulated genes were a gap junction protein (connexin-31), tight junction proteins (ZO1 and occludin), and a key component in the TGFβ signaling pathway (Smad2).

Moreover, Saravanan *et al.* analyzed the differential expression of glycosyltransferases in healing mouse corneas using glycogene microarrays.^25^ They found 11 enzymes to be upregulated, namely glycosyltransferases, beta3GalT5, T-synthase, GnTIVb, and 19 enzymes to be downregulated, namely GnTIII and sialyltransferases, in healing mouse corneas compared to normal uninjured corneas.

Retinal fibrosis is the common pathophysiological mechanism in blinding retinal diseases, such as age-related macular degeneration (AMD), diabetic retinopathy, and proliferative vitreoretinopathy (PVR). Using an Affymetrix human genome microarray, Hollborn *et al.* studied differentially expressed genes between retinas of two PVR patients and seven postmortem normal retinas.^26^ They found upregulation of 80 genes, namely encoding nuclear and cell cycle-related proteins, extracellular secretory proteins, cytosolic signaling proteins, and extracellular matrix proteins. The HGF and heparin-binding EGF-like growth factor genes were also expressed in PVR retinas, but not in control retinas.

In addition, Asato *et al.* studied the gene expression profile of three eyes with PVR-epiretinal membranes and two eyes with secondary epiretinal membranes.^27^ They found 1116 non-redundant clusters representing individual genes expressed in PVR-epiretinal membranes, and 799 clusters representing the genes expressed in secondary epiretinal membranes. Their results support that PVR-epiretinal membranes represent an aberrant form of the wound healing response in the retina, with an increased expression of genes involved in cell adhesion and proliferation.

There have been several genomic studies to date, but most of them have been carried in animals or small cohorts of patients (Table 1). Future research is thus needed to validate these results in large longitudinal human studies. One of the major hurdles has been the lack of availability of human tissues for biomarker research. Effective detailed clinical phenotyping and biobanking of large cohorts of patients will therefore be crucial to study putative biomarkers in fibrotic eye diseases compared to healthy controls. Another exciting prospect in the future will be to use new advances in genomics, namely next-generation whole genome sequencing like RNA-Seq, to develop a customized gene chip in ocular fibrosis.

**Table 1.** Potential novel biomarkers in ocular fibrosis and wound healing

*Biomarkers*	*References*	*Techniques*	*Species and Tissues*	*Results*
Tissue genomics	Popp *et al.*^6^	Microarray	Rabbit conjunctiva and Tenon's capsules after GFS	315 genes, namely encoding serum amyloid A-3 protein, IL-1 beta, alpha-1-acid glycoprotein, cathepsin K, MMP-9, neutrophil granules matrix glycoprotein SGP28, ceruloplasmin, lumican, lysozyme C, and fibronectin
	Esson *et al.*^7^	Microarray	Rat blebs after GFS	923 genes, namely encoding TGFβ, CTGF, FGF, IGF, matrix metalloproteinases, collagens, vimentin, and fibronectin
	Mahale *et al.*^8^	RT^2^ Profiler PCR Array	7 human Tenon's capsules of failed Ahmed valves	39 genes, including *CTGF, THBS1, SERPINE1, THBS2, COL3A1, MMP3, and IL1A* genes
	Varela *et al.*^23^	Microarray	Rat corneas after Excimer PRK	73 genes, including growth factors (*VEGF, FGF, IGF-I*), proteases (*PAI-1, PAI-2A*), and protease inhibitors (*TIMP-2, TIMP-3*)
	Burton *et al.*^14,15^	Microarray Real-time quantitative PCR	45 trachomatous trichiasis patients 386 trachomatous trichiasis patients	Squamous metaplasia (keratins, SPRR), proinflammatory cytokine production (*IL1*β, *CXCL5*, *S100A7*), and tissue remodeling (*MMP7*, *MMP9*, *MMP12*, and *HAS3*)
	Razzaque *et al.^17–20^*	Real-time quantitative PCR	10 patients with ocular cicatricial pemphigoid	Upregulated *m-CSF*, *CTGF*, *HSP47*, and *MIF* genes
	Cao *et al.*^24^	cDNA microarrays	Healing mouse corneas	37 genes upregulated and 27 genes downregulated, namely encoding ICAM-1, macrophage inflammatory proteins, SOCS, IL-10 receptor, galectin-7, connexin-31, ZO1 and occludin, and Smad2
	Saravanan *et al.*^25^	Glycogene microarrays	Healing mouse corneas	11 enzymes upregulated and 19 enzymes downregulated, including glycosyltransferases, beta3GalT5, T-synthase, GnTIVb, GnTIII, and sialyltransferases
	Hollborn *et al.*^26^	Affymetrix human genome microarray	2 human retinas of PVR patients	80 genes upregulated, namely encoding nuclear and cell cycle related, extracellular secretory, cytosolic signaling, and extracellular matrix proteins, HGF, and HB-EGF
	Asato *et al.*^27^	PCR-amplified cDNA library	3 human eyes with PVR-epiretinal membranes	1116 gene clusters, namely related to metabolism, cell adhesion, cytoskeleton, and signaling
Serum and tear biomarkers	Tezel *et al.*^33^	Linear ion trap mass spectrometry	111 patients with POAG	63 proteins, including AIF, CREB-binding protein, ephrin type-A receptor, and huntingtin protein
	Chong *et al.*^34^	Tear cytokine profile using multiplex bead assay	61 glaucoma patients	Increased MCP-1 level
	Dunmire *et al.*^35^	Liquid chromatography– Mass spectrometry	Rhesus macaques sera after laser-induced retinal injury	19 proteins, including phosphoglycerate kinase 1, keratin 18, Lewis alpha-3-fucosyltransferase, and ephrin receptor A2
	Scott *et al.*^36^	Liquid chromatography– Mass spectrometry	Rabbit sera after laser-induced retinal injury	4 candidate autoantigens, namely dihydropyrimidinase-related protein 2, fructose-bisphosphate aldolase C, chaperonin-containing T-complex polypeptide 1 subunit zeta, and pyruvate kinase isozyme
	Kierny *et al.*^37^	Mass spectrometry, phage-display	Rabbit sera after laser-induced retinal injury	Antibodies against 4 peptides derived from putative biomarkers; GBB5 retinal protein
Proteomics	Saccà *et al.*^42^	Antibody microarray method	Aqueous humour of 14 POAG patients	13 proteins, including apolipoprotein B, apolipoprotein E, vasodilator-stimulated phosphoprotein, heat shock 60 kDa protein, heat shock 90 kDa protein, myogenin, myogenic factor 3, myotrophin, ankyrin, ubiquitin fusion degradation 1-like, phospholipase C beta 1, phospholipase C gamma 1, and albumin
	Anshu *et al.*^43^	Liquid chromatography– Mass spectrometry	Aqueous humour of 11 patients with glaucoma tube implants	13 proteins, including gelsolin, plasminogen, angiotensinogen, apolipoprotein A-II, beta-2-microglobulin, dickkopf-3, pigment epithelium-derived factor, RIG-like 7–1, afamin, fibronectin 1, apolipoprotein A-I, activated complement C4 protein, and prothrombin
	Rosenfeld *et al.*^44^	Liquid chromatography– Mass spectrometry	Aqueous humour of 20 patients with glaucoma implants	718 proteins, splice variants or isoforms
	Mandal *et al.*^45^	Liquid chromatography– Mass spectrometry	Rabbit detached retina	18 proteins, including vimentin, tubulin β-2C, fragments of α-enolase, fructose-bisphosphate, aldolase A, ATP synthase subunit β, mitochondrial creatine kinase, N-terminal fragments of albumin, prohibitin, and transducin-β1
	Yu *et al.*^46^	Mass spectrometry	24 human vitreous samples from RRD patients with PVR	Upregulated alpha2-HS-glycoprotein, alpha1B-glycoprotein, complement components, and hemopexin; Downregulated opticin precursors and actin family members
	Yu *et al.*^47^	Liquid chromatography– Mass spectrometry	24 PVR patients with RRD	Upregulated transferrin, albumin precursor, alpha2-HS-glycoprotein, alpha1B-glycoprotein, serpins family, and complement components; Downregulated tubulin, pyruvate kinase 3, enolase, and GAPDH
Metabolomics	Karamichos *et al.*^52^	Mass spectrometry	Human keratoconus cell lines	Elevated lactate levels, lactate/malate, and lactate/pyruvate ratios; Reduced arginine levels and glutathione/oxidized glutathione ratio
	Li *et al.*^54^	Mass spectrometry	17 human vitreous samples from RRD and PVR patients	31 metabolites, including L-carnitine, urea, phenylpyruvate, cyromazine, hypoxanthine, citrate, glycerate, ascorbate, and 2-Oxoglutarate
	Osborn *et al.*^55^	Liquid chromatography– Mass spectrometry	26 patients with neovascular AMD and fibrovascular tissue	94 metabolic features, namely acetylphenylalanine, glycocholic acid, vitamin D-related metabolites, phenylalanine, tyrosine, glutamine, and aspartate
	Agudo-Barriuso *et al.*^56^	Mass spectrometry	Rat optic nerve injury	27 metabolites (between control and 14 days) and 36 metabolites (between 24 h and 14 days), linked to translation, oxidative stress, glucose and tricarboxylic acid cycle and apoptosis
Clinical phenotyping	Kon *et al.*^57^ Asaria *et al.*^58^	Univariate and multivariate logistic regression analysis	140 patients having a primary vitrectomy for rhegmatogenous retinal detachment	Risk factors for PVR: Preoperative PVR, aphakia, and high vitreous protein levels
	Rajak *et al.*^59^	Logistic regression analysis	1300 patients with trachomatous trichiasis in Ethiopia	Risk factors for recurrence: Preoperative major trachomatous trichiasis (>5 trichiatic lashes), preoperative entropic lashes compared to misdirected/metaplastic lashes, and age over 40 years
Noninvasive Imaging	Farid *et al.*^65^	Second-harmonic generation confocal microscopy	Rabbit corneal fibrosis after excimer laser surface ablation	High cell density and alignment of intracellular actin filaments with collagen fiber bundles
	Teng *et al.*^67^	Multiphoton imaging and second-harmonic generation microscopy	1 patient with penetrating corneal injury	Irregularly arranged collagen fibers and lack of collagen fibers within the corneal wound
	Kawana *et al.*^70^	Anterior segment OCT	38 filtering blebs in 31 patients	Successful blebs showed a large internal fluid-filled cavity, an extensive hyporeflective area, and thicker bleb walls
	Singh *et al.*^71,72^	Anterior segment OCT	78 filtering blebs in 55 patients	Successful blebs displayed thickening of the bleb wall
	Siriwardena *et al.*^73^	Laser flare meter	131 trabeculectomy patients	Increased anterior chamber flare and inflammation
	Wolff *et al.*^75^	Spectral domain OCT	15 eyes with neovascular AMD	Branching retinal tubulation network emanating from a fibrovascular scar
	Coscas *et al.*^76^	Spectral domain OCT	38 patients with fibrovascular PED	Homogeneous hyperreflectivity consistent with fibrous tissue

GFS, glaucoma filtration surgery; PCR, polymerase chain reaction; PVR, proliferative vitreoretinopathy; POAG, primary open-angle glaucoma; RRD, rhegmatogenous retinal detachment; AMD, age-related macular degeneration; OCT, optical coherence tomography; PED, pigment epithelial detachment.

### Serum and tear biomarkers

As tissue biopsy can lead to scarring, serum and tear biomarkers represent a less invasive alternative to tissue biopsy in fibrosis (Table 1). The enhanced liver fibrosis (ELF) test is a clinical-grade serum test that could be used as a biomarker of overall fibrosis in liver fibrosis^28^ and systemic sclerosis.^29^ FibroMeters are blood tests that display a high overall diagnostic accuracy in staging and quantification of liver fibrosis, and are useful for patient follow-up.^30^ The serum levels of miR-29a are also significantly associated with patients with liver fibrosis,^31^ and hypertrophy and fibrosis in patients with hypertrophic cardiomyopathy.^32^

For the anterior segment of the eye, Tezel *et al.* used liquid chromatography and linear ion trap mass spectrometry to compare the sera of 111 patients with primary open-angle glaucoma (POAG) and 49 healthy controls.^33^ They found 106 proteins to be increased in the glaucomatous sera and 63 proteins to be present in only the glaucomatous sera. They validated their results and identified four molecules (apoptosis-inducing factor, CREB-binding protein, ephrin type-A receptor, huntingtin protein) with higher serum enzyme-linked immunosorbent assay titers in the POAG patients. Chong *et al.* also determined the tear cytokine profile using a multiplex bead assay in 61 glaucoma patients and 29 normal subjects, and found that the eyes at risk of scarring in the early postoperative period had significantly increased MCP-1 level.^34^

Serum biomarkers have been studied in more detail for the posterior segment of the eye, namely following laser-induced retinal injuries. Dunmire *et al.* used liquid chromatography–tandem mass spectrometry and found 19 proteins to be significantly increased in the sera of Rhesus macaques after laser-induced retinal injury.^35^ Four proteins (phosphoglycerate kinase 1, keratin 18, Lewis alpha-3-fucosyltransferase, and ephrin receptor A2) showed significant differences at both 4 hours and 1 day after laser injury, followed by a decrease to baseline levels by the third day. Scott *et al.* also identified four autoantigens in the sera of rabbits after minimally invasive and grade II laser-induced retinal injuries using liquid chromatography/tandem mass spectrometry.^36^ The four candidate autoantigens were dihydropyrimidinase-related protein 2, fructose-bisphosphate aldolase C, chaperonin-containing T-complex polypeptide 1 subunit zeta, and pyruvate kinase isozyme.

In addition, Kierny *et al.* generated recombinant antibodies against putative biomarkers of retinal injury in rabbit sera following laser-induced retinal injury.^37^ They generated single-chain variable fragment antibodies against four peptides derived from putative biomarkers of laser-induced retinal injury using phage display. One antibody against the retinal protein, guanine nucleotide-binding protein beta 5 (GBB5), was carried further to demonstrate a method to characterize antibodies generated from peptide fragments identified by mass spectrometry of serum samples.

### Proteomics

Proteomics is gaining increasing interest in the field of eye research due to recent advances in protein chemistry, mass spectrometry, and bioinformatics^38^ (Table 1). Several groups are developing potential proteomic biomarkers in idiopathic pulmonary fibrosis^39^ and liver fibrosis.^40,41^ In the eye, Saccà *et al.* compared the aqueous humour proteome of 14 POAG patients to that of normal controls using the antibody microarray method.^42^ They found the levels of 13 proteins to be significantly increased in the aqueous humour of POAG patients. These proteins are involved in inflammation, delivery of cholesterol to cells, muscle cell differentiation, stress response, and signal transduction, and might reflect the damage occurring to the trabecular meshwork and to the anterior chamber endothelia in glaucoma.

Anshu *et al.* also identified 13 proteins to be significantly higher in the aqueous humour of 11 patients with a glaucoma tube implant using liquid chromatography–mass spectrometry.^43^ These proteins play a role in oxidative stress, apoptosis, inflammation, and immunity, and their presence in the aqueous humour suggests that glaucoma tube implants cause either a breach in the blood–aqueous barrier or chronic trauma, increasing the influx of oxidative, apoptotic, and inflammatory proteins. In addition, Rosenfeld *et al.* studied the aqueous humour of 20 patients with different glaucoma operations.^44^ They found the aqueous humour protein concentrations to be ten-fold in the Ahmed and Baerveldt eyes and five-fold in the trabeculectomy and Ex-PRESS eyes. They also identified 718 unique proteins, splice variants or isoforms using liquid chromatography–tandem mass spectrometry.

Retinal detachment leads to cellular remodeling of the retina. Mandal *et al.* reported that 18 proteins were differentially expressed between detached rabbit retina and controls using liquid chromatography–tandem mass spectrometry.^45^ The proteins identified were involved in a wide range of processes, including cell metabolism, cell structure, mitochondrial function, and phototransduction, and might play an important role in the wound response of the retina after its detachment and its subsequent ability to recover following surgical reattachment.

Using sodium dodecyl sulfate–polyacrylamide gel electrophoresis and reverse-phase liquid chromatography–tandem mass spectrometry, Yu *et al.* also found 48 overlapping proteins in the vitreous of 24 patients with rhegmatogenous retinal detachment (RRD) and PVR.^46^ The authors used GeneGo MetaCore (Version 6.6; GeneGo, Carlsbad, CA) for the enrichment flow analysis. They found inflammation to be an important GeneGo network and identified the complement and coagulation cascade as the essential pathway. Yu *et al.* also carried out a vitreous proteomic analysis in 24 PVR patients with RRD using two-dimensional nano liquid chromatography coupled with tandem mass spectrometry.^47^ They identified 102 PVR-specific proteins and proposed that kininogen 1 could become a potential candidate biomarker as it was specifically detected in both the vitreous and corresponding serum samples.

### Metabolomics

Metabolomics is a relatively new technology that measures the set of metabolites that make up the metabolome of a cell or tissue,^48^ and there has been growing interest in using metabolomics to identify clinically relevant biomarkers^49^ (Table 1). Several metabolic products have been reported as biomarkers of fibrosis in nonalcoholic fatty liver disease.^50^ Alterations in metabolic pathways have also been reported in idiopathic pulmonary fibrosis and measurement of these metabolites could be used as future diagnostic and prognostic biomarkers.^51^

Keratoconus is a corneal ectasia that is associated with corneal scarring, and altered cellular metabolism has been linked to promoting the fibrotic phenotype and scarring in the cornea. Karamichos *et al.* identified that several of the metabolic pathways that were significantly different between human keratoconus cells and human corneal keratocytes were related to oxidative stress, and that human keratoconus cells expressed elevated lactate levels, lactate/malate and lactate/pyruvate ratios, and reduced arginine levels and glutathione/oxidized glutathione ratio.^52^

PVR is the major cause of failure of retinal detachment surgery and is caused by contraction of fibrotic membranes on the epiretinal surface of the neurosensory retina.^53^ Using reversed-phase liquid chromatography–quadrupole time-of-flight mass spectrometry, Li *et al.* identified 31 metabolites as potential biomarkers in 17 vitreous samples of PVR patients.^54^ Inflammation, proliferation, and energy consumption were the three major disturbed biological processes involved in PVR development.

Osborn *et al.* also performed a metabolome-wide association study of 26 patients with neovascular AMD and fibrovascular tissue using liquid chromatography and Fourier transform mass spectrometry.^55^ They found 94 unique metabolic features to be significantly different between patients with neovascular AMD and fibrovascular tissue compared to healthy controls.

In addition, Agudo-Barriuso *et al.* identified 27 metabolites to discriminate between control and 14 days after rat optic nerve injury, using gas chromatography/mass spectrometry and liquid chromatography/mass spectrometry techniques.^56^ Enrichment analysis showed alterations in the amino acid, carbohydrate, and lipid metabolism that were further linked to translation, oxidative stress, energy (glucose and tricarboxylic acid cycle), and apoptosis through ceramide pathways.

### Clinical phenotyping

**Figure 3.**
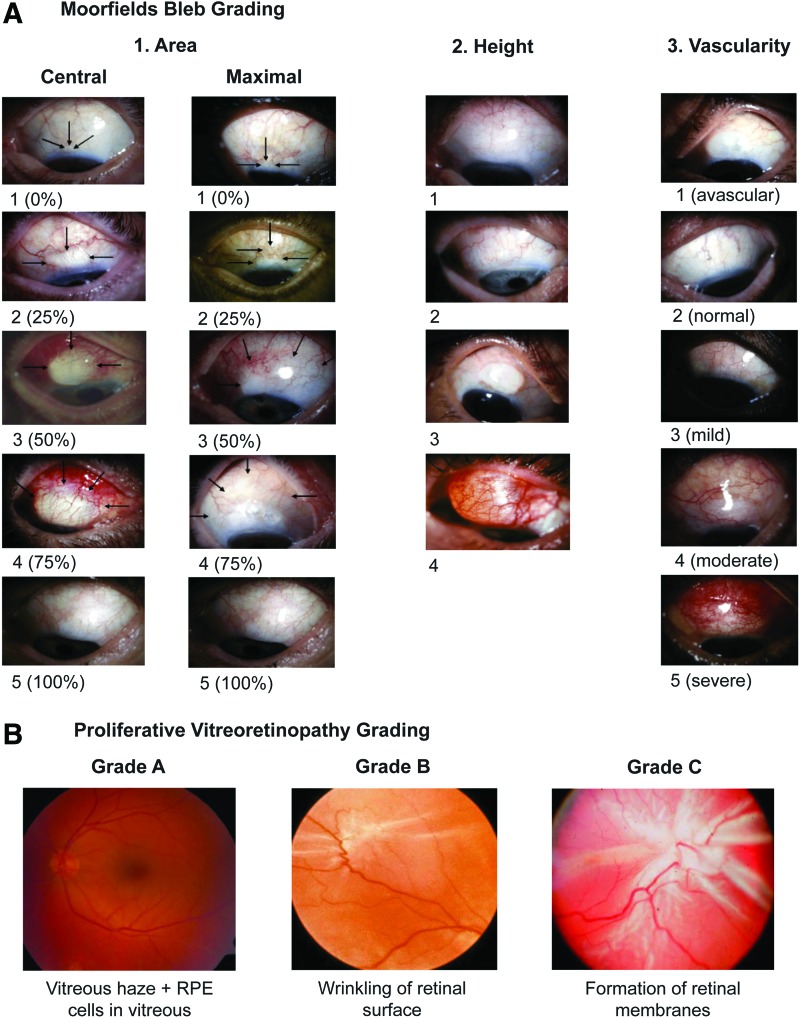
Detailed clinical phenotyping of patients alongside effective biobanking of tissues from large patient cohorts will be critical for future biomarker research in ocular fibrosis. **(A)** Glaucoma blebs are graded with respect to area [scale 1–5], height [scale 1–4], and vascularity [scale 1–5]. **(B)** The severity of proliferative vitreoretinopathy is graded as Grade A [vitreous haze and RPE cells in vitreous], Grade B [wrinkling of the edges of the retinal tear or inner retinal surface], or Grade C [formation of retinal membranes]. To see this illustration in color, the reader is referred to the web version of this article at www.liebertpub.com/wound

Clinical phenotyping is another critical aspect of personalized medicine and several groups have studied potential clinical models to predict the risk of scarring and fibrosis in the eye (Fig. 3 and Table 1). Kon *et al.* carried out a univariate and multivariate logistic regression analysis on 140 patients with RRD.^57^ They found that the significant risk factors associated with the development of PVR were preoperative PVR, aphakia, and high vitreous protein levels. The authors also constructed two statistical models (clinical factors only and clinical factors combined with vitreous protein level) to predict the probability of developing postoperative PVR and to identify the high-risk patients that might benefit from pharmacological antifibrotic therapies.^57,58^

Moreover, trachoma is the most common infectious cause of blindness worldwide and causes trichiasis, leading to conjunctival scarring and visual loss. Lid rotation surgery is the mainstay of treatment for TT, but the risk of recurrence is high. Rajak *et al.* carried out a 2-year follow-up study of 1300 patients with TT in Ethiopia.^59^ The authors found that recurrence was associated with specific clinical features such as major TT preoperatively (>5 trichiatic lashes), preoperative entropic lashes compared to misdirected/metaplastic lashes, and age over 40 years.

### Noninvasive imaging

**Figure 4.**
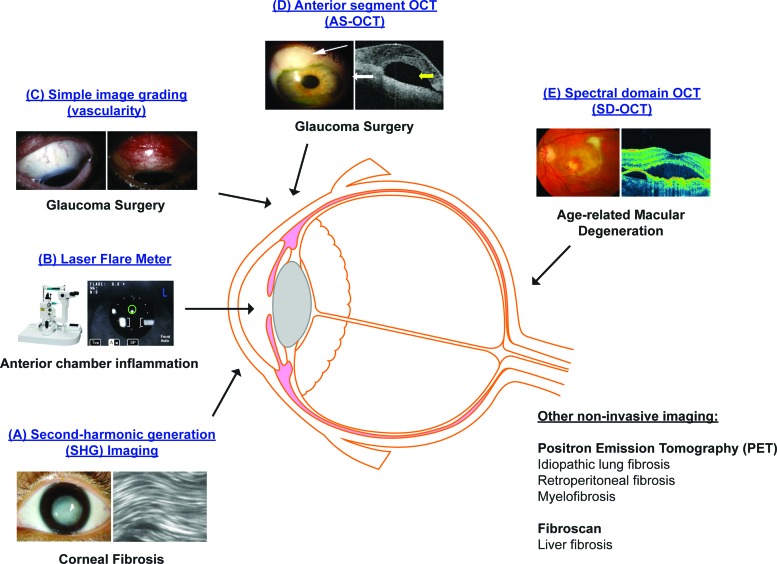
High-resolution noninvasive *in vivo* imaging represents a promising new technique to help stratify patients with different fibrotic eye diseases: **(A)** SHG imaging; **(B)** Laser flare meter; **(C)** Simple image grading (vascularity); **(D)** AS-OCT; **(E)** SD-OCT. SHG, second-harmonic generation; AS-OCT, anterior segment optical coherence tomography; SD-OCT, spectral domain optical coherence tomography. To see this illustration in color, the reader is referred to the web version of this article at www.liebertpub.com/wound

High-resolution real-time *in vivo* imaging represents a promising new technique to help stratify patients in fibrosis and wound healing (Fig. 4 and Table 1). Positron Emission Tomography (PET) is a functional noninvasive imaging technique, whereby the concentration of the biologically active tracer, fluorodeoxyglucose, corresponds to tissue metabolic activity. Several research groups have studied the use of PET scans to monitor idiopathic lung fibrosis,^60^ retroperitoneal fibrosis,^61^ and myelofibrosis.^62^ FibroScan is also an ultrasound-based transient elastography technique and is currently the most reliable noninvasive method to assess liver stiffness and fibrosis, as an alternative to liver biopsy.^63,64^

In the eye, several groups have shown that high-resolution, high-contrast second-harmonic generation imaging provides a sensitive means to detect corneal fibrosis after excimer laser surface ablation and corneal injury.^65–67^ The corneal wound was associated with high cell density and alignment of intracellular actin filaments with collagen fiber bundles,^65^ and irregularly arranged collagen fibers.^67^ The authors thus suggested that high-resolution *in vivo* imaging could be used in the future to assess the effects of antifibrotic therapy on corneal wound healing after refractive surgery or corneal injury.

Simple image grading, particularly of redness indicating inflammation, is a very good indicator of impending failure of glaucoma filtration surgery.^68^ Other research groups have also investigated the use of anterior segment optical coherence tomography (AS-OCT) to predict the surgical failure and scarring after glaucoma drainage surgery.^69–72^ AS-OCT was used to assess different bleb characteristics, including total bleb height, bleb cavity, bleb wall thickness, scleral flap thickness, and patency of the internal ostium. Features of successful blebs were thickening of the bleb wall,^70,71^ a large internal fluid-filled cavity, and an extensive hyporeflective area compared to unsuccessful blebs.^70^

In addition, recent cataract surgery is a risk factor for failure of glaucoma filtration surgery. Using the Kowa FM-500 laser flare meter that measures the level of aqueous humour protein noninvasively in 131 patients undergoing trabeculectomy and 148 patients undergoing cataract surgery, Siriwardena *et al.* found that the anterior chamber flare was more prolonged after cataract surgery than after trabeculectomy.^73^ The release of lens crystallins and lens epithelial cells might upregulate the production of fibrogenic cytokines in the aqueous humour of patients after cataract surgery.^74^ The authors thus suggested that anterior chamber flare could be used as a surrogate biomarker of anterior chamber inflammation and help guide the timing of trabeculectomy in relation to cataract surgery.

Different research groups have also studied spectral-domain optical coherence tomography (SD-OCT) as a potential future tool to diagnose and follow-up patients with fibrovascular retinal diseases. Using SD-OCT, Wolff *et al.* found a branching retinal tubulation network emanating from a fibrovascular scar among 15 eyes of patients with neovascular AMD.^75^ Coscas *et al.* also studied 38 patients with fibrovascular pigment epithelial detachment (PED) and identified a homogeneous hyperreflectivity pattern consistent with fibrous tissue using SD-OCT.^76^

## Summary

There have been significant advances in the use of genomics, proteomics, metabolomics, clinical phenotyping, and high-resolution *in vivo* imaging techniques to help identify and stratify the groups of patients at risk of scarring in different fibrotic eye diseases (Table 1). However, most of the studies to date have been carried out in animals or small cohorts of patients, and future research is thus needed to validate these results in large longitudinal human studies. Effective detailed clinical phenotyping, including high-resolution imaging and biobanking of tissues from large patient cohorts, will also be crucial to compare putative biomarkers in ocular fibrosis to healthy controls.

Take-Home Messages

• There is a large unmet clinical need for reliable biomarkers in ocular and systemic fibrosis.
• There have been significant advances in genomics, proteomics, metabolomics, and high-resolution imaging to help stratify the risk of scarring in fibrotic eye diseases.
• Future research is needed to validate these results in large longitudinal human studies.
• Effective detailed clinical phenotyping, including imaging and biobanking of tissues from large patient cohorts, will be critical to compare putative biomarkers in ocular fibrosis to healthy controls.
• Successful translation of these biomarkers will ultimately require a strong collaboration between academics, pharmaceutical, and biotech companies.
• An exciting future prospect will be to use next-generation whole genome sequencing, like RNA-Seq, to develop a customized gene chip in ocular fibrosis.

As tissue biopsy can itself induce scarring in the eye, there has been growing interest in developing less invasive biomarkers such as serum or tear biomarkers and high-resolution *in vivo* imaging techniques. Another exciting future prospect will be to use new advances in genotyping, namely next-generation whole genome sequencing like RNA-Seq, to develop a customized gene chip in ocular fibrosis. We believe that a strong collaboration between academics, pharmaceutical, and biotech companies will also be critical for successful translation of these biomarkers to benefit patient care. The ultimate goal in the future will be to apply diagnostic and therapeutic biomarkers and to develop a more stratified approach in antifibrotic ocular therapeutics and personalized visual health.

## About the Authors

**Cynthia Yu-Wai-Man, MBBS, FRCOphth,** is an NIHR BRC Francis Crick Institute Clinical Research Fellow and is based at the UCL Institute of Ophthalmology and the Francis Crick Institute. Her research is focused on identifying novel targets to prevent scarring in the eye and on translational antifibrosis eye research. Dr. Yu-Wai-Man is also working on potential novel biomarkers to develop a more personalized and stratified approach in antifibrotic ocular therapeutics.

**Peng Tee Khaw, PhD, FRCS, FRCP, FRCOphth, FRCPath, FRSBiol, FARVO, FMedSci**, is Professor of Glaucoma and Wound healing and Director of the NIHR Biomedical Research Centre at Moorfields Eye Hospital NHS Foundation Trust and UCL Institute of Ophthalmology. For over 25 years, he has pioneered new surgical techniques and new treatments to prevent scarring and to promote regeneration of tissues after ocular surgery and disease.

## Abbreviations and Acronyms

AIF: apoptosis-inducing factor
AMD: age-related macular degeneration
AS-OCT: anterior segment OCT
CCL11: chemokine (C-C motif) ligand 11
CEACAM5: carcinoembryonic antigen-related cell adhesion molecule 5
COL3A1: collagen type III alpha 1
CREB: cyclic AMP-responsive element-binding protein
CTGF: connective tissue growth factor
CXCL5: chemokine (C-X-C motif) ligand 5
CXCR4: chemokine (C-X-C motif) receptor 4
DNA: deoxyribonucleic acid
EGF: epidermal growth factor
ELF: enhanced liver fibrosis
ERM: epiretinal membrane
FGF: fibroblast growth factor
GAPDH: glyceraldehyde-3-phosphate dehydrogenase
GBB5: guanine nucleotide-binding protein beta 5
GFS: glaucoma filtration surgery
HAS3: hyaluronan synthase 3
HGF: hepatocyte growth factor
HSP47: heat shock protein 47
ICAM: intercellular adhesion molecule
IGF: insulin-like growth factor
IL: interleukin
MCP-1: monocyte chemoattractant protein 1
m-CSF: macrophage colony-stimulating factor
MIF: migration inhibitory factor
MMP: matrix metalloproteinase
OCP: ocular cicatricial pemphigoid
OCT: optical coherence tomography
PAI: plasminogen activator inhibitor
PCR: polymerase chain reaction
PED: pigment epithelial detachment
PET: positron emission tomography
POAG: primary open-angle glaucoma
PVR: proliferative vitreoretinopathy
RNA-Seq: RNA sequencing
RRD: rhegmatogenous retinal detachment
S100A7: S100 calcium-binding protein A7
SAGE: serial analysis of gene expression
SD-OCT: spectral domain OCT
SDS-PAGE: sodium dodecyl sulfate–polyacrylamide gel electrophoresis
SHG: second-harmonic generation
SOCS: suppressor of cytokine signaling
SPARCL1: secreted protein, acidic and rich in cysteine-like 1
TGFβ: transforming growth factor beta
THBS1: thrombospondin 1
THBS2: thrombospondin 2
TIMP: tissue inhibitor of metalloproteinase
TT: trachomatous trichiasis
VEGF: vascular endothelial growth factor
αSMA: alpha smooth muscle actin
